# An integrative taxonomic approach reveals a new species of *Eranthis* (Ranunculaceae) in North Asia

**DOI:** 10.3897/phytokeys.140.49048

**Published:** 2020-03-04

**Authors:** Andrey S. Erst, Alexander P. Sukhorukov, Elizaveta Yu. Mitrenina, Mikhail V. Skaptsov, Vera A. Kostikova, Olga A. Chernisheva, Victoria Troshkina, Maria Kushunina, Denis A. Krivenko, Hiroshi Ikeda, Kunli Xiang, Wei Wang

**Affiliations:** 1 Central Siberian Botanical Garden, Siberian Branch of Russian Academy of Sciences, 101 Zolotodolinskaya Str., 630090, Novosibirsk, Russia Central Siberian Botanical Garden, Siberian Branch of Russian Academy of Sciences Novosibirsk Russia; 2 Tomsk State University, 36 Lenin Ave., 634050, Tomsk, Russia Tomsk State University Tomsk Russia; 3 Lomonosov Moscow State University, Leninskie Gory 1/12, 119234, Moscow, Russia Lomonosov Moscow State University Moscow Russia; 4 South-Siberian Botanical Garden, Altai State University, 61 Lenin Ave., Barnaul, 656049, Russia Altai State University Barnaul Russia; 5 Siberian Institute of Plant Physiology and Biochemistry, Siberian Branch of Russian Academy of Sciences, 132 Lermontov Str., 664033, Irkutsk, Russia Siberian Institute of Plant Physiology and Biochemistry, Siberian Branch of Russian Academy of Sciences Irkutsk Russia; 6 The University Museum, The University of Tokyo, Hongo 7-3-1, Bunkyo-ku, Tokyo 113-0033, Japan The University of Tokyo Tokyo Japan; 7 State Key Laboratory of Systematic and Evolutionary Botany, Institute of Botany, Chinese Academy of Sciences, 100093, Beijing, China Institute of Botany, Chinese Academy of Sciences Beijing China; 8 University of Chinese Academy of Sciences, 19 Yuquan Road, Beijing, 100049, China University of Chinese Academy of Sciences Beijing China

**Keywords:** Biochemistry, cytology, integrative taxonomic approach, morphology, phylogeny, Ranunculales, Russia

## Abstract

A new endemic species, *Eranthis tanhoensis***sp. nov.**, is described from the Republic of Buryatia and Irkutsk Province, Russia. It belongs to Eranthis section Shibateranthis and is morphologically similar to *E. sibirica* and *E. stellata*. An integrative taxonomic approach, based on cytogenetical, molecular and biochemical analyses, along with morphological data, was used to delimit this new species.

## Introduction

The genus *Eranthis* L. (Ranunculaceae) consists of eight to ten species distributed in southern Europe and temperate Asia (Lee et al. 2012; Park et al. 2019). Most species have narrow distributions and only one European species, *E. hyemalis* (L.) Salisb., has been widely cultivated in gardens and become naturalised in Britain (Boens 2014) and North America (Parfitt 1997). *Eranthis* are perennial herbs with tuberous rhizomes, basal long-petiolate leaves with the blades divided into several or many palmate segments (leaflets) that are entire or lobate; unbranched scapes carrying a solitary, bisexual and actinomorphic flower supported by three verticillate leaf-like bracts forming an involucre; (4–)5–8 yellow, white or pink, caducous sepals; 5–10(–15) yellow or white, bifid petals shorter than sepals; nectaries located at the middle or upper part of the petals; > 10 stamens; and 3–10 follicles with several smooth seeds in each fruitlet (Parfitt 1997). All species are early-blooming plants, with anthesis from March to May (depending on the altitude), but *E. hyemalis* has been found at full anthesis in mid-January in gardens (Sukhorukov, pers. obs. in Mainz, Germany, 2019 and Leiden, Netherlands, 2020).

On the basis of morphology, the genus has been divided into two sections: E. sect. Eranthis and E. sect. Shibateranthis (Nakai) Tamura (Tamura 1987). The type section is characterised by annual tubers, yellow sepals and emarginate or slightly bilobate upper petal margins without swellings (nectaries), whereas the members of section Shibateranthis have long-lived tubers, white sepals and bilobate or forked petal margins with swellings (Tamura 1995). Molecular phylogenetic analysis. based on nrITS and chloroplast *trn*L-*trn*F interspacer region, supports the subdivision of the genus into these sections (Park et al. 2019). Furthermore, they are geographically separated, with section Eranthis occurring in Europe (*E. hyemalis*) and SW & W Asia (*E. cilicica* Schott & Kotschy, *E. longistipitata* Regel) and section Shibateranthis distributed in temperate N & E Asia (*E. albiflora* Franch., *E. byunsanensis* B.Y.Sun, *E. lobulata* W.T.Wang, *E. pinnatifida* Maxim., *E. pungdoensis* B.U.Oh, *E. sibirica* DC. and *E. stellata* Maxim.: Park et al. 2019). Two additional species with yellow sepals, *E. bulgarica* (Stef.) Stef. (Stefanoff 1963) and *E. iranica* Rukšāns & Zetterl. (Rukšāns and Zetterlund 2018), have been described from Bulgaria and Iran, respectively, but have not yet been included in molecular analysis.

Recent studies have revealed the genetic diversity, phylogeny and presumed origin of some narrowly distributed Korean and Japanese species with further conclusions about their taxonomic status (Lee et al. 2012; Oh and Oh 2019). The taxonomic and genetic diversity of *Eranthis* in the Asiatic part of Russia is insufficiently studied. To date, only two species have been found in Russia: *E. sibirica* and *E. stellata* (both belonging to sect. Shibateranthis) from South Siberia and Far East Russia (Malyshev 2005). High genetic polymorphism of *E. sibirica* across populations near Baikal Lake was discovered only recently (Protopopova et al. 2015) and this fact has inspired us to conduct a new study of *Eranthis* in the Asiatic part of Russia.

The aim of the present study was to investigate the morphological, molecular, biochemical and cytogenetic heterogeneity of the Baikal populations to determine whether any undescribed species were present there. The relationship between *E. sibirica*, *E. stellata* and a new species, described and named below as *Eranthis tanhoensis* Erst, sp. nov. is explored here.

## Materials and methods

### Plant material

More than 300 herbarium specimens were collected during field investigations in the Republics of Khakassia and Buryatia and the Irkutsk Province during 2018 and 2019. Fieldwork was conducted during different seasons to observe the species in both their flowering and fruiting stages. The specimens were deposited in the E and NS herbaria (herbarium abbreviations according to Thiers 2019+). Revision of herbarium materials was undertaken in the herbaria at IRK, LE, MW, NS, NSK, PE, VBGI and VLA. Drawings of the new species, *Eranthis tanhoensis*, are based on images of the type specimen (NS-0000948!) and paratype (NS-0000949!). The flowering and fruiting times and habitats are provided as cited on the collectors’ labels. Maps of records were made with SimpleMappr (http://www.simplemappr.net). Conservation analysis was performed using criteria from the International Union for the Conservation of Nature (IUCN 2019). The Extent of Occurrence (EOO) and Area of Occupancy (AOO) of each species were estimated using GeoCat (Bachman et al. 2011).

### Molecular analysis

We sampled 15 individuals of *E. tanhoensis* and six of *E. sibirica*. Two individuals of *E. stellata* and one each of *E. pinnatifida* and *E. longistipitata* were also included. The details of the samples are presented in Suppl. material 1: Table S1. Six nuclear and plastid DNA regions (ITS, *trn*L-F, *trn*H-*psb*A, *rps16*, *mat*K and *rbc*L) were included in the molecular analysis. Total genomic DNA was extracted from silica gel-dried leaves or herbarium specimens using DNeasy Mini Plant Kits (Tiangen Biotech, Beijing, China) following the protocol specified by the manufacturer. Sequencing reactions were conducted using BigDyeTM Terminators (Applied Biosystems Inc., Foster City, CA, USA). Sequences were read using an automated ABI 3730xl DNA Analyzer. Geneious v8.0.4 (Kearse et al. 2012) was used to evaluate the chromatograms for base confirmation and to edit contiguous sequences. We first used the Maximum Likelihood (ML) method to perform non-parametric bootstrap analyses for each DNA region in RAxML v7.0.4 (Stamatakis 2006). No significant bootstrap support for conflicting nodes was evident amongst individual DNA regions (here considered to exceed 70%) and the six-locus datasets were therefore combined for subsequent analyses. Phylogenetic analyses of the combined dataset were conducted using ML and Bayesian Inference (BI) methods. RAxML was conducted with the GTR + Γ substitution model for each region with the fast bootstrap option using 1000 replicates. BI analysis was conducted in MrBayes v3.2.1 (Ronquist et al. 2012). Data partitioning and nucleotide substitution models were determined using PartitionFinder 2.1.1 (Lanfear et al. 2016). Two independent analyses, consisting of four Markov Chain Monte Carlo chains were run, sampling one tree every 1000 generations for 10 million generations. Runs were completed when the average standard deviation of split frequencies reached 0.01. The stationarity of the runs was assessed using Tracer v1.6 (Rambaut et al. 2014). After removing the burn-in period samples (the first 25% of sampled trees), a majority rule (> 50%) consensus tree was constructed.

### Morphological analysis

The morphology of vegetative and reproductive structures was examined on well-developed specimens. For numerical analysis, 25 specimens at flowering and 25 specimens at fruiting stages were examined for each species (more than 150 specimens altogether). For each species, we studied different populations from across the range, including populations from the type localities of *E. stellata* and *E. sibirica*. As *E. stellata* often does not produce basal leaves at flowering, we studied this character in a limited number of samples. The morphological characters were measured using AxioVision 4.8 software (Carl Zeiss, Munich, Germany).

The missing values in the original data table were restored using multidimensional linear regression, in accordance with recommendations of Myers (2000) and Lee and Carlin (2010). A one-way analysis of variance (ANOVA), according to Chambers et al. (1992), was used to identify the distinguishing morphometric features of each species. The differences were considered significant at P-value < 0.05. As multiple statistical testing was performed, the calculated P-value was adjusted using the procedure proposed by Benjamini and Hochberg (1995). The principal component analysis was used to visualise the distribution of the analysed individuals over the space of morphometric characters. This method was employed only for those characters that displayed significant intergroup differences, according to the results of the ANOVA. For scale adjustment, the logarithmic transformation of data was used. The results of the principal component analysis were visualised using the Factoextra package (Kassambara and Mundt 2017).

### Cytogenetic analysis

Somatic chromosomes were studied in root tip cells. Tubers were germinated in wet moss at ~15 °C for 2–4 weeks. Newly formed 1–2 cm long roots were excised and pretreated in a 0.5% colchicine solution for 2–3 h at 15 °C. Roots were fixed in a mixture of 96% ethanol and glacial acetic acid (3:1). Root tips were stained with 1% aceto-haematoxylin and the squash method was employed for investigation of the karyotype (Smirnov 1968).

Chromosomes were counted in 50–100 mitotic cells for each population. Mitotic metaphase chromosome plates were observed using an Axio Star microscope (Carl Zeiss, Munich, Germany) and photographed using an Axio Imager A.1 microscope (Carl Zeiss, Germany) with AxioVision 4.7 software (Carl Zeiss, Germany) and AxioCam MRc5 CCD–camera (Carl Zeiss, Germany) at 1000× magnification in the Laboratory for Ecology, Genetics and Environmental Protection (Ecogene) of the National Research Tomsk State University. KaryoType software (Altinordu et al. 2016) was used for karyotyping, whereas Adobe Photoshop CS5 (Adobe Systems, USA) and Inkscape 0.92 (USA) were used for image editing. Karyotype formulae were based on measurements of mitotic metaphase chromosomes taken from photographs. The measurements were performed on 5–10 metaphase plates. The symbols used to describe the karyotypes followed those of Levan et al. (1964): m = median centromeric chromosome with arm ratio of 1.0–1.7 (metacentric chromosome); sm = submedian centromeric chromosome with arm ratio of 1.7–3.0 (submetacentric chromosome); st = subterminal centromeric chromosome with arm ratio of 3.0–7.0 (subtelocentric chromosome); t = terminal centromeric chromosome with arm ratio of 7.0–∞ (acrocentric chromosome); T = chromosome without obvious short arm, i.e. with arm ratio of ∞.

### Flow cytometry

Flow cytometry with propidium iodide (PI) staining was used to determine the absolute DNA content. The relative DNA content in the nucleus (C-value) in representatives of three *Eranthis* species – *E. stellata*, *E. sibirica* and *E. tanhoensis* from different populations, was determined in this study. In total, more than 70 samples from 15 populations were studied (see Suppl. material 1: Table S1). Silica gel-dried leaf material (0.5–1.0 cm^2^) was chopped with a sharp razor blade in a 1 ml cold nuclei extraction buffer composed of 50 mM Hepes, 10 mM sodium metabisulphite, 10 mM MgCl_2_, 0.5% polyvinylpyrrolidone, 0.1% bovine serum albumin, 0.3% Tween20, 0.2% Triton X-100, 50 μg/ml RNase, 1 μg/ml β-mercaptoethanol and 50 μg/ml propidium iodide (PI). The samples were filtered through 50 μm nylon membranes into sample tubes and incubated in the dark at 4 °C for 15 min. Samples were measured using a Partec CyFlow PA flow cytometer equipped with a green laser, at 532 nm wavelength. The absolute nuclear DNA content, the 2C-value according to Greilhuber et al. (2005), was calculated as the ratio of the mean fluorescence intensity of the nuclei of the sample to that of an external standard multiplied by the total nuclear DNA content of the standard. The possible effect of secondary metabolites on the binding of the intercalating dye was evaluated by measuring the fluorescence of *Allium fistulosum* L. leaf samples prepared as described above, but with the addition of the supernatant from *Eranthis* samples, centrifuged without PI. The samples were measured three times at 10 min intervals. If no variation in the average values of the detection channels was observed for the *A. fistulosum* peak, the effect of secondary metabolites was considered negligible.

The 1Cx-value (monoploid DNA content *sensu*Greilhuber et al. 2005) was calculated by dividing the 2C-value by the ploidy level of the species. The species, used as external standards, were *Zamioculcas zamiifolia* Engl., 2С = 48.35 pg and *Vicia faba* L. ‘Inovec’ 2С = 26.90 pg (Doležel et al. 1992; Skaptsov et al. 2016). We used the Statistica 8.0 software (StatSoft, Inc.), Flowing Software 2.5.1 (Turku Centre for Biotechnology) and CyView software (Partec, GmbH) for data analyses. Flow cytometry was performed at the Laboratory for Bioengineering of the South-Siberian Botanical Garden, Altai State University (Barnaul, Russia).

### High-performance liquid chromatography (HPLC) analysis of individual phenolic compounds in ethanol leaf extracts

In order to determine the composition of phenolic compounds, air-dried plant material was mechanically ground to obtain a homogenous powder and then samples of ~0.2 g were extracted three times using 70% aqueous ethanol solution for 30 min in a water bath at 72 °C. Next, the combined extract was concentrated in porcelain dishes to 5 ml. The solutions were filtered and stored at 4 °C until analysis. Analysis of phenolic components was performed using an Agilent 1200 HPLC system equipped with a diode array detector and a ChemStation system for the collection and processing of chromatographic data (Agilent Technology, Palo Alto, CA, USA). The separation was performed on a Zorbax SB-C18 column (5 µm, 4.6 × 150 mm) at 25 °C. The methanol content of the mobile phase in an aqueous solution of phosphoric acid (0.1%) varied from 50–52% over 56 min (van Beek 2002). The eluent flow rate was 1 ml/min. Detection wavelengths were 255, 270, 340 and 360 nm. Groups of phenolic substances were identified by their spectral characteristics (Bate-Smith 1962; Mabry et al. 1970). For identification of the phenolic components in plant extracts, standard samples of salicylic and chlorogenic acids, quercetin, kaempferol, orientin (Sigma-Aldrich Chemie GmbH, Munich, Germany), gentisic and caffeic acids (Serva Heidelberg, Germany), hyperoside and vitexin (Fluka Chemie AG, Buchs, Switzerland) were used. The samples were analysed twice.

## Results and discussion

### Molecular phylogenetic analysis

**Figure 1.**
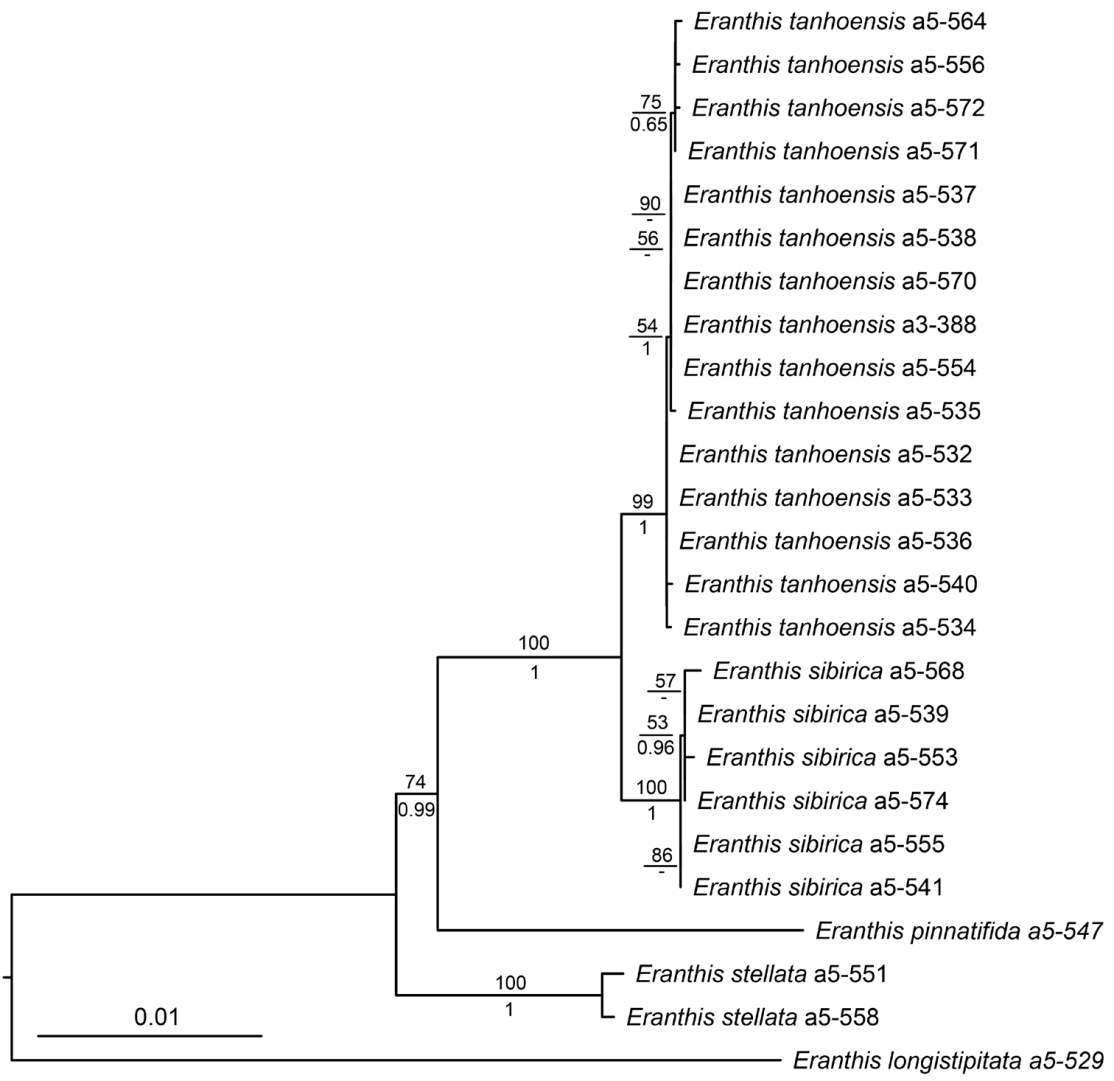
ML tree inferred from the combined cpDNA and ITS data. The numbers above branches are bootstrap values (BS > 50%) and numbers under branches are Bayesian posterior probabilities (PP > 0.50).

Bayesian and ML analyses of the combined dataset produced highly consistent topologies. *Eranthis sibirica* and the new species *E. tanhoensis* formed a sister clade of that of *E. pinnatifida*. The monophyly of each species, *E. tanhoensis* sp. nova, *E. sibirica* and *E. stellata*, was strongly supported (Fig. 1).

### Morphological analysis

The morphological analysis revealed that *E. sibirica* was not homogeneous across its distribution area. We compared 41 characters to distinguish *E. sibirica*, *E. tanhoensis* and *E. stellata* (Suppl. material 1: Table S2). The basal and involucral leaves in *Eranthis* spp. undergo changes at fruiting and, for this reason, the lengths of all leaves, their segments and segment lobes were measured both at the flowering and fruiting stages. In Suppl. material 1: Table S2, an asterisk (*) indicates the characters used in the numerical analysis. An ANOVA was conducted only for quantitative characteristics. As basal leaves are often absent at the time of flowering and there were no samples with basal leaves in herbarium collections, there were limited data on these characteristics of *E. stellata*.

**Figure 2.**
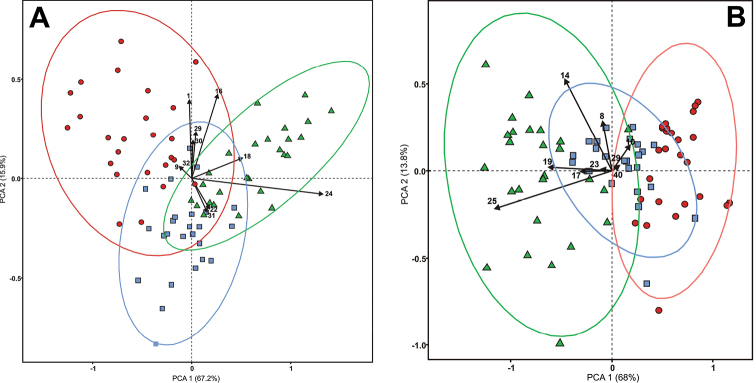
Scatter point diagram in the space of the first two main components for *Eranthis sibirica* (red dots), *Eranthis tanhoensis* (green triangles) and *Eranthis stellata* (blue squares) **A** at flowering and **B** at fruiting stages. Ellipses enclose the regions of the space that contain each of the plant species with a 95% probability (95% confidence ellipses).

The ANOVA of morphometric characters showed significant differences amongst the studied species in characters (1), (9), (16), (18), (22), (24), (29), (30), (31) and (32) at the flowering stage and (6), (10), (14), (17), (19), (23), (25), (40) and (41) at the fruiting stage (Suppl. material 1: Tables S3, S4). In total, significant differences amongst the species were found in 10 out of 15 morphometric parameters measured at flowering and in 9 out of 13 parameters at fruiting. The principal component analysis revealed that the first two main components accounted for 83.1% and 81.8% of the variance in the entire data array of the parameters measured at flowering and fruiting, respectively and showed the best species discrimination. The highest variability of morphometric characters was found at flowering in *E. sibirica* (Fig. 2A) and at fruiting in *E. tanhoensis* (Fig. 2B). As signified by the directions of the vectors indicating the gradients in the character values, at flowering, *E. sibirica* differed from *E. tanhoensis* by having lower values for characters (18), (22), (24) and (31) and a higher value for character (9). At fruiting, *E. sibirica* was characterised by having lower values for parameters (19), (17), (23) and (25) and higher values for parameters (10), (40) and (29), in comparison with those of *E. tanhoensis. E. sibirica* differed from *E. stellata* by having higher values for characters (1), (16), (29), (30) and (32) at flowering and (10) and (14) at fruiting. The pattern of overlap between the species differed between flowering and fruiting plants. For instance, *E. tanhoensis* was reliably distinguished from *E. sibirica* only at fruiting (the ellipses enclosing the samples did not overlap; Fig. 2B). In addition to numerical parameters, the new species was also distinguished by qualitative characters.

### Cytogenetic analysis

The karyotypes of three related species, *E. sibirica*, *E. tanhoensis* and *E. stellata*, were investigated (Table 1), those of *E. sibirica* and *E. tanhoensis* being studied for the first time. The chromosomes of each species were medium or large in size (from 5 to 11–12 µm) and belonged to the R-type (Langlet 1932). The vouchers are listed in Suppl. material 1: Table S1.

**Table 1.** Chromosome numbers (2*n*), ploidy level (n*x*), karyotype formulas, and C-values (C ± SD) of the three studied *Eranthis* species.

Voucher number	Species	Voucher information	2*n*	n*x*	Karyotype formulae	2C±SD, pg	1Cx±SD, pg
1	*E. sibirica*	Republic of Khakassia, Bolshoi On river	28	4*x*	2*n* = 20m (2 sat) + 2m/sm + 6 sm	38.83±1.03	9.71±0.26
2	*E. sibirica*	Irkutsk Province, Kuitun river	28	4*x*	2*n* = 20m (2 sat) + 2m/sm + 6 sm	38.19 ± 0.28	9.55 ± 0.14
3	*E. sibirica*	Irkutsk Province, Slyudyanka river	42	6*x*	2*n* = 30m + 12sm (2 sat)	55.75±0.28	9.23±0.14
4	*E. sibirica*	Irkutsk Province, Burovschina river	42	6*x*	2*n* = 30m + 12sm (2 sat)	55.76±0.47	9.27±0.23
5	*E. sibirica*	Irkutsk Province, Utulik river	42	6*x*	2*n* = 30m + 12sm (2 sat)	55.31±0.45	9.22±0.25
6	*E. tanhoensis*	Irkutsk Province, Mamai river	14	2*x*	2*n* = 10m (2sat) + 4sm	24.88±0.54	12.44±0.27
7	*E. tanhoensis*	Republic of Buryatia, Duliha river	14	2*x*	2*n* = 10m (2sat) + 4sm	24.97±0.43	12.49±0.22
8	*E. tanhoensis*	Republic of Buryatia, Tolbazikha river	14	2*x*	2*n* = 10m (2sat) + 4sm	24.77±0.52	12.38±0.26
9	*E. tanhoensis*	Irkutsk Province, Malye Mangaly river	14	2*x*	2*n* = 10m (2sat) + 4sm + 0–8B	24.15±0.11	12.07±0.06
10	*E. tanhoensis*	Irkutsk Province, Semirechka river	14	2*x*	2*n* = 10m (2sat) + 4sm	25.31±0.15	12.41±0,29
11	*E. tanhoensis*	Republic of Buryatia, Osinovka river (Tanhoi village)	14	2*x*	2*n* = 10m (2sat) + 4sm	25.11±0.32	12.56±0.16
12	*E. tanhoensis*	Republic of Buryatia, Mishiha river	14	2*x*	2*n* = 10m (2sat) + 4sm + 0–4B	25.25±0.15	12.07±0.07
13	*E. tanhoensis*	Republic of Buryatia, Shestipalikha river	14	2*x*	2*n* = 10m (2sat) + 4sm	25.53±0.18	12.77±0.09
14	*E. stellata*	Primorsky Krai, Vladivostok, Studencheskaya railway station	16	2*x*	2*n* = 16 = 10m + 4sm (2sat) + 2t	31.76±0.61	15.88±0.31
15	*E. stellata*	Primorsky Krai, Malaya Sedanka river	16	2*x*	2*n* = 16 = 10m + 4sm (2sat) + 2t	31.88±0.67	15.94±0.34
16	*E. stellata*	Primorsky Krai, “13^th^ km” railway station	16	2*x*	2*n* = 16 = 10m + 4sm (2sat) + 2t	–	–
17	*E. stellata*	Primorsky Krai, Russkiy Island	16	2*x*	2*n* = 16 = 10m + 4sm (2sat) + 2t	28.47±0.46	14.23±0.23

**Figure 3.**
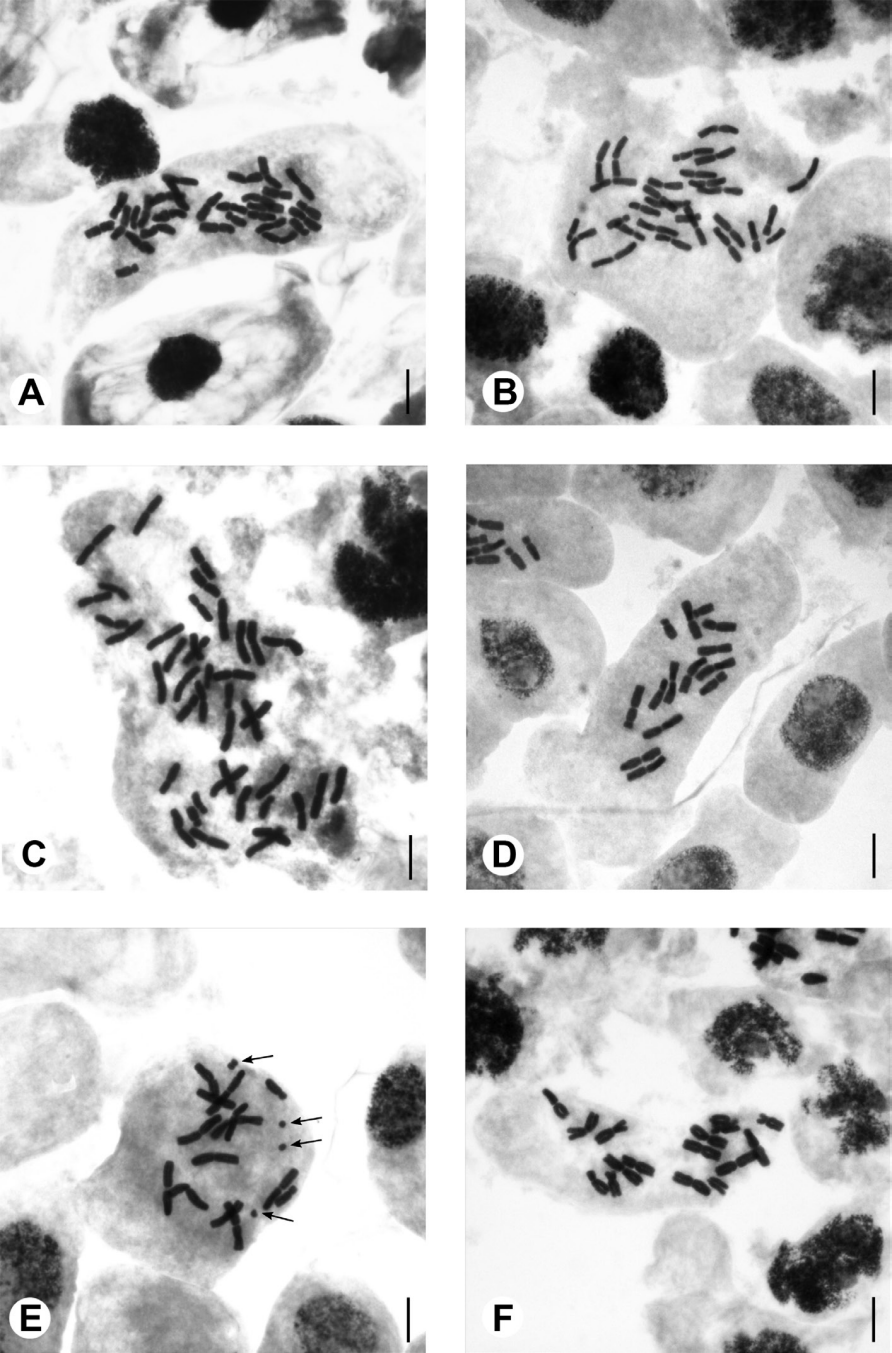
Mitotic metaphase chromosomes. **A***Eranthis sibirica* (voucher 1 in Table 1), 2*n* = 28 **B***Eranthis sibirica* (voucher 2), 2*n* = 28 **C***Eranthis sibirica* (voucher 3), 2*n* = 42 **D***Eranthis tanhoensis* (voucher 11), 2*n* = 14 **E***Eranthis tanhoensis* (voucher 9), 2*n* = 14 + 0–8 B (arrows point at B chromosomes) **F***Eranthis stellata* (voucher 14), 2*n* = 16. Scale bars: 10 µm.

*Eranthis sibirica*. Two cytotypes, with basic chromosome number *x* = 7, were revealed. *Eranthis sibirica* from the Republic of Khakassia (1) and Irkutsk Province (2) were tetraploid with 2*n* = 4*x* = 28 (Fig. 3A, B). Three populations from the Irkutsk Province (3, 4 and 5) were hexaploid with 2*n* = 6*x* = 42 (Fig. 3C). Metacentric and submetacentric chromosome types were present in all examined *E. sibirica* specimens. The karyotype formula of tetraploid plants was 2*n* = 20m(2sat) + 2m/sm + 6sm and 2n = 30m + 12sm(2sat) in hexaploid plants. No B chromosomes were identified in this species.

*Eranthis tanhoensis*. We determined the chromosome numbers in specimens of eight populations of *E. tanhoensis*. All plants studied were diploid, with 2*n* = 2*x* = 14 (Table 1 and Fig. 3D, E). Metacentric and submetacentric types of chromosomes were found (Fig. 3D, E). The two populations examined (9, 12) were characterised by the presence of B chromosomes. The maximum number of B chromosomes appeared to be eight (9). B chromosomes in this species were represented by two types: small (2.3–2.5 μm) metacentrics and dot-shaped 1.3–1.5 μm long chromosomes, which were obviously acrocentric. The karyotype formula of *E. tanhoensis* was 2*n* = 10m (2sat) + 4sm + 0–8B.

*Eranthis stellata*. In all four studied populations of *E. stellata*, the basic chromosome number was *x* = 8. This species was diploid with 2*n* = 2*x* = 16, which is typical of the genus (Table 1; Fig. 3F). Five pairs of chromosomes were metacentric, two pairs were submetacentric and one pair was acrocentric (Fig. 3F). The karyotype formula of *E. stellata* was 2*n* = 10m + 4sm (2sat) + 2t. No B chromosomes were observed in this species.

The basic chromosome number *x* = 8 has been reported for the entire genus *Eranthis* (Langlet 1932; Kurita 1955; Tak and Wafai 1996; Gömürgen 1997; Yuan and Yang 2006; Kim et al. 2011; Marhold et al. 2019). Our results are consistent with previously published data (Yuan and Yang 2006), with insignificant differences in the karyotype formula. However, we showed, for the first time, that *E. sibirica* and *E. tanhoensis* are distinguished from other species of the genus by the basic chromosome number *x* = 7. Such differences in basic chromosome numbers (*x* = 7 and *x* = 8) have been found in some other genera of Ranunculaceae, for example, *Anemone* L. and *Ranunculus* L. (Rice et al. 2015). Our results regarding the chromosome numbers in *E. sibirica* (2*n* = 28 and 2*n* = 42) differed from the data reported by other researchers for this species (2*n* = 32: Krogulevich (1976) or 2*n* = 16: Gnutikov et al. (2016, 2017)). *Eranthis tanhoensis* was found to have 2*n* = 14. Based on the incongruence of the chromosome data with previous and recent analyses, we assume that some populations of *E. sibirica* and *E. tanhoensis* may have diverse cytotypes. Both species clearly differed from *E. stellata* by the absence of acrocentrics. All three species were characterised by five metacentrics and two submetacentrics per monoploid chromosome set.

### Flow cytometry

The average absolute DNA content of hexaploid samples of *E. sibirica* was 2C = 55.33 ± 0.52 pg and that of tetraploid samples was 2C = 38.19 ± 0.28 pg. In diploid *E. tanhoensis*, the average absolute DNA content was 2C = 25.02 ± 0.28 pg. The average absolute DNA content of diploid *E. stellata* was 2C = 31.47 ± 0.46 pg. The monoploid DNA content of the *E. sibirica* cytotypes was similar: 1Cx = 9.55 ± 0.14 pg in tetraploids and 1Cx = 9.25 ± 0.20 pg in hexaploids. The monoploid DNA content of *E. tanhoensis* was 1Cx = 12.49 ± 0.16 pg and that of *E. stellata* was 1Cx = 15.77 ± 0.20 pg.

Tetraploids and hexaploids of *E. sibirica* exhibited insignificant differences in DNA content (9.25 pg for 6*x* and 9.55 for 4*x*), whereas diploids of *E. tanhoensis* showed a higher 1Cx level (12.49 pg), which may indicate a relatively ancient diversification of these species. Data on the 1Cx level of *E. stellata* (15.77 pg) indicated the independent or parallel evolution of genome size in this species. According to flow cytometry, variations in 1Cx levels between diploid samples of *E. tanhoensis* and hexaploids and tetraploids of *E. sibirica* were in accordance with the hypothesis of genome downsizing in polyploid flowering plants (Leitch and Bennett 2004).

### HPLC analysis of individual phenolic compounds

Phenolic compounds are often used in chemotaxonomic studies owing to their wide distribution in plants, structural diversity and chemical stability (Braunberger et al. 2015; Radušienė et al. 2018). They have also been reported as promising chemotaxonomic markers for Ranunculaceae (Hao 2018). However, data on the significance of these substances for the taxonomy of *Eranthis* is still insufficient. Only a few studies of the phytochemical characteristics of certain *Eranthis* species, considered as medicinal plants, have been published (Djafari et al. 2018; Hao 2018; Watanabe et al. 2003, 2019).

**Figure 4.**
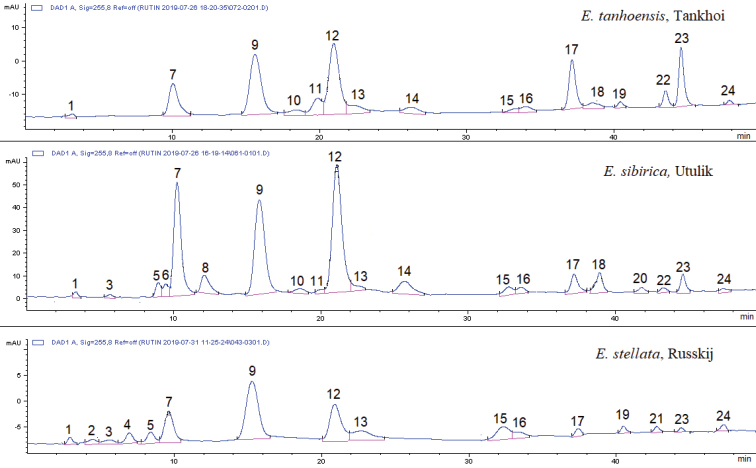
HPLC chromatograms of 70% water-ethanol extracts of *Eranthis* leaves detected by HPLC-DAD at 255 nm. The X-axis displays the retention time, min; Y-axis – the detector signal in optical density units. The identified peaks are **1.** chlorogenic acid, **2.** gentisic acid, **3.** caffeic acid, **5.** orientin, **8.** vitexin, **10.** hyperoside, **11.** salicylic acid, **19.** quercetin and **24.** kaempferol.

Twenty four phenolic compounds were detected in 70% ethanol extracts of plant leaves of the three *Eranthis* species (*E. sibirica*, *E. stellata* and *E. tanhoensis*) using HPLC (Fig. 4). Phenolic acids (chlorogenic, gentisic, caffeic and salicylic acids), flavonols (quercetin, kaempferol and hyperoside) and flavones (orientin and vitexin) were identified amongst these compounds. All three species were very similar in the composition of the phenolic compounds extracted from their leaves; however, there were specific compounds for each taxon. The common compounds present in all studied plants were chlorogenic acid, phenolic acids (Fig. 4, peak 3: t_R_, min = 10.0, λ_max_, nm = 250, 290 sh, 335; peak 12: t_R_, min = 20.9, λ_max_, nm = 240, 290 sh, 335 and peak 23: t_R_, min = 44.3, λ_max_, nm = 255, 300, 330) and flavonols (Fig. 4, peak 9: t_R_, min = 15.1, λ_max_, nm = 255, 360 and peak 15: t_R_, min = 32.7, λ_max_, nm = 270, 310, 365). Almost all plants contained kaempferol, phenolic acids (Fig. 4, peak 14: t_R_, min = 25.4, λ_max_, nm = 255, 300 and peak 16: t_R_, min = 34.5; λ_max_, nm = 250, 290, 330) and flavonols (Fig. 4, peak 13: t_R_, min = 22.3, λ_max_, nm = 255, 360 and peak 18: t_R_, min = 38.7, λ_max_, nm = 255, 305, 360). *Eranthis sibirica* leaves contained about 15 to 20 phenolic compounds, whereas, in *E. stellata* leaves, their number varied from 16 to 18. Phenolic compounds were less diverse in *E. tanhoensis* leaves than in leaves of other species, whose numbers varied from 13 to 16 substances.

The chromatographic profile of *E. sibirica* differed from that of *E. tanhoensis* in the presence of caffeic acid, orientin, vitexin and flavone (peak 6: t_R_, min = 9.4, λ_max_, nm = 270, 310) in 70% ethanol leaf extracts (Fig. 4). Caffeic acid, orientin and flavone (peak 6) were generally absent from leaves of *E. tanhoensis*, whereas vitexin was found in some samples in trace amounts. The leaves of *E. tanhoensis* from almost all the studied populations contained quercetin, which was not detected in *E. sibirica*. Distinguishing compounds in leaf extracts of *E. stellata* were gentisic acid, phenolic acid (Fig. 4, peak 4: t_R_, min = 7.1, λ_max_, nm = 250, 300) and flavone (Fig. 4, peak 21: t_R_, min = 42,2; λ_max_, nm = 210, 310), which were absent from the two other species. Vitexin, hyperoside and salicylic acids were not found in *E. stellata* leaves. All samples of *E. stellata* contained orientin and caffeic acid, which were characteristic of *E. sibirica* and quercetin, which was typical of *E. tanhoensis*.

### Taxonomy

The analysis of the data presented above allowed us to distinguish a new species from specimens previously identified as *E. sibirica*.

#### Eranthis tanhoensis

Taxon classificationPlantaeRanunculalesRanunculaceae

Erst sp. nov.

BF662E60-CE41-58B8-A5E9-83D4AF687719

urn:lsid:ipni.org:names:77206949-1

Figs 5 , 6A–D , 7B

##### Type.

Russia, Republic of Buryatia, Kabansky district, Osinovka River near Tanhoi village, 51°33'06.2"N, 105°05'34.7"E, 458 m a.s.l., 01 May 2019, *A.S. Erst*, *D.A. Krivenko*, & *O.A. Chernysheva s.n.* (holotype, NS-0000948!, isotypes TK, IRK, E).

##### Description.

*Herb* perennial, 12.0–23.0 cm long at flowering and 18.0–40.0 cm long at fruiting. *Tubers* subglobose, not or slightly branching, 1.2–3.3 cm diam., producing thin fibrous roots. *Basal leaf* single, long-petiolate, green; petioles 5.0–6.0 cm long at flowering and 23–25 cm at fruiting; blades 2.5–3.8 × 2.5–3.5 cm at flowering and 7.5–12 × 7.5–12 cm at fruiting, deeply palmately divided into 5 segments (maximum length of segment dissection 2.3 cm at flowering (3.5 cm at fruiting)); leaf blade segments rounded or widely rhombic, 0.8–2.5 × 0.4–1.8 cm at flowering (1.7–8.5 × 1.2–7.5 cm at fruiting), unlobed or dissected into 1–2 lobes at both flowering and fruiting stages; segment of basal leaves with 5–19 acute teeth at apex at flowering, 6–25 teeth at fruiting. *Involucre* present, 1.1–5.5 cm diam. at flowering (7–11 cm at fruiting stage); involucral bracts (cauline leaf) sessile, laciniate, similar to basal leaf, divided into 5 trifid leaf-like segments (maximum length of segment dissection is 1.6 cm at flowering (4.0 cm at fruiting)); segments rounded or widely rhombic, 1.1–3.0 × 0.5–2.5 cm at flowering (3.3–6.4 × 1.4–5.3 cm at fruiting), unlobed or dissected into 2 lobes both at flowering and fruiting stages; each segment with 5–21 teeth (at both flowering and fruiting stages), acute at the apex. *Pedicels* 0.5–1.5 cm long, elongated in fruiting (3.5–5.5 cm long), densely covered with papillate and large hemispherical trichomes. *Flowers* bisexual, actinomorphic, solitary, erect, 2–4 cm diam. *Sepals* 4–7, deciduous in fruit, white or light pink at margin, flat, narrowly obovate or elliptic, 1.1–2.6 × 0.5–1.3 cm. *Petals* 5–15 × 0.6–0.8 cm long, bicoloured, white, tubular, two-lipped with bilobate or forked lips, each lobe of abaxial lip acute at the apex and with globular yellow swellings (nectaries: Fig. 7B). Stamens 36–45, 0.7–1.1 cm long; filaments filiform, white; anthers white. Follicles 3–10, 0.8–1.4 cm long, on short (0.3–0.5 mm) stalks, divergent towards the end of fruiting; stylodium 0.1–0.3 mm long, straight or slightly curved.

##### Notes.

Turczaninow (1842) described the species *E. uncinata* Turcz., growing at higher altitudes and distinguished from *E. sibirica* by the number of petals (5–6, not strictly 5), by the shape of the stylodium (recurved rather than straight), smaller flowers and more dissected leaf blades. However, our studies have shown that these morphological characters are variable and all variations can be found both in the foothill and alpine plants. Shipchinskiy (1937) merged *E. uncinata* with *E. sibirica*. However, he described two varieties: E. sibirica DC. var. nuda Schipcz. with glabrous pedicels (= E. sibirica var. sibirica) and E. sibirica DC. var. glandulosa Schipcz. with glandular-pubescent pedicels. These varieties were not validly published under ICN Article 39.1 (Turland et al 2018). Nakai (1937) attributed *E. sibirica* and *E. uncinata* to the genus *Schibateranthis* Nakai (≡ Eranthis sect. Schibateranthis (Nakai) Tamura).

##### Affinity.

**Figure 5.**
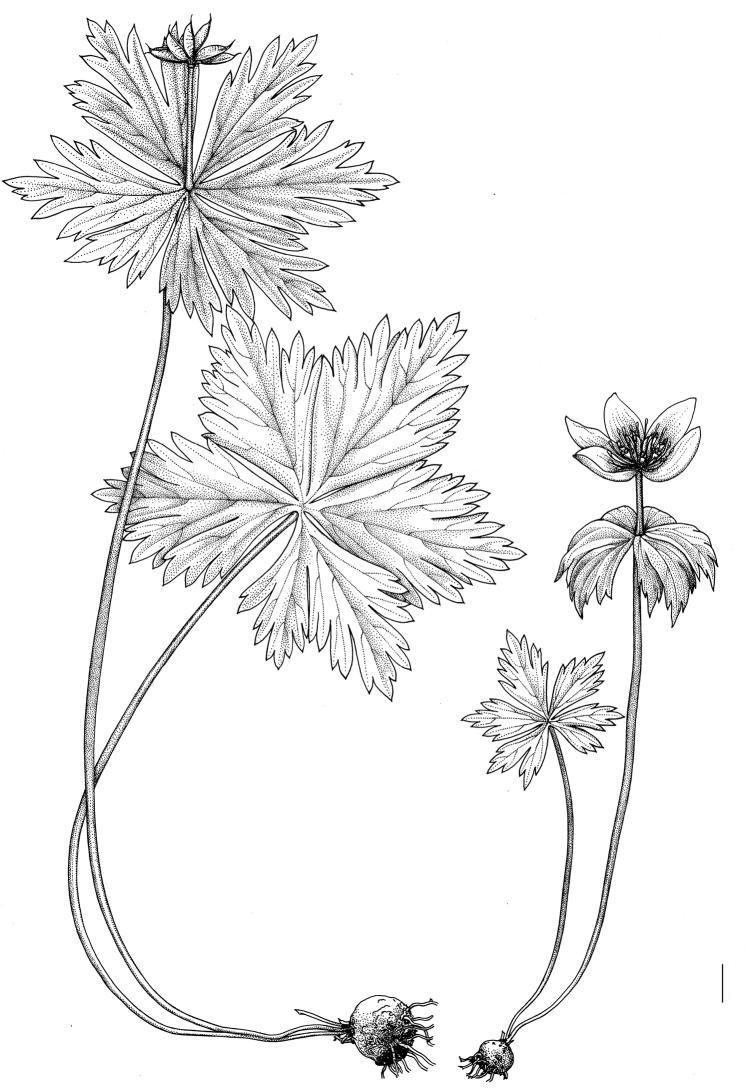
General habit of *Eranthis tanhoensis*. Scale bar: 1 cm.

**Figure 6.**
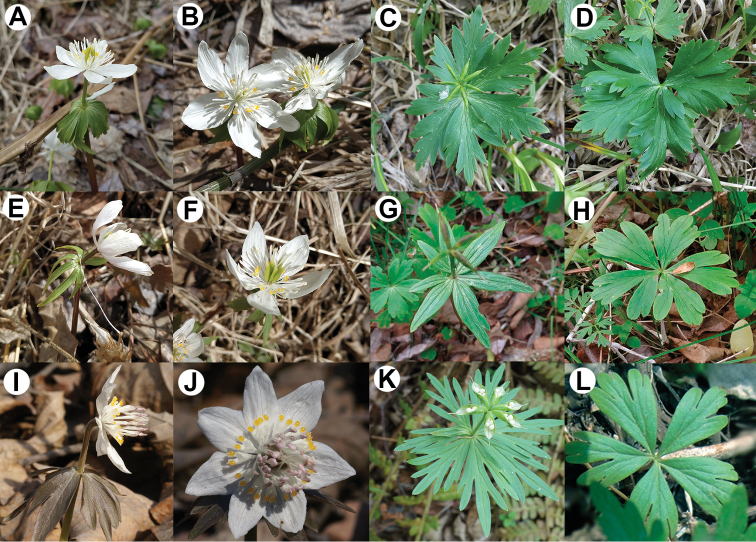
Morphological differences amongst **A–D***Eranthis tanhoensis***E–H***Eranthis sibirica*; and **I–L***Eranthis stellata***A, E, I** flower position **B, F, J** flowers **C, G, K** involucral bracts and follicles **D, H, L** basal leaves.

**Figure 7.**
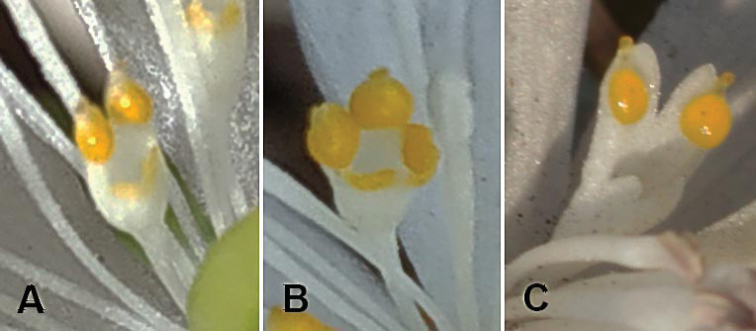
Petals. **A***Eranthis sibirica***B***Eranthis tanhoensis***C***Eranthis stellata*.

**Figure 8.**
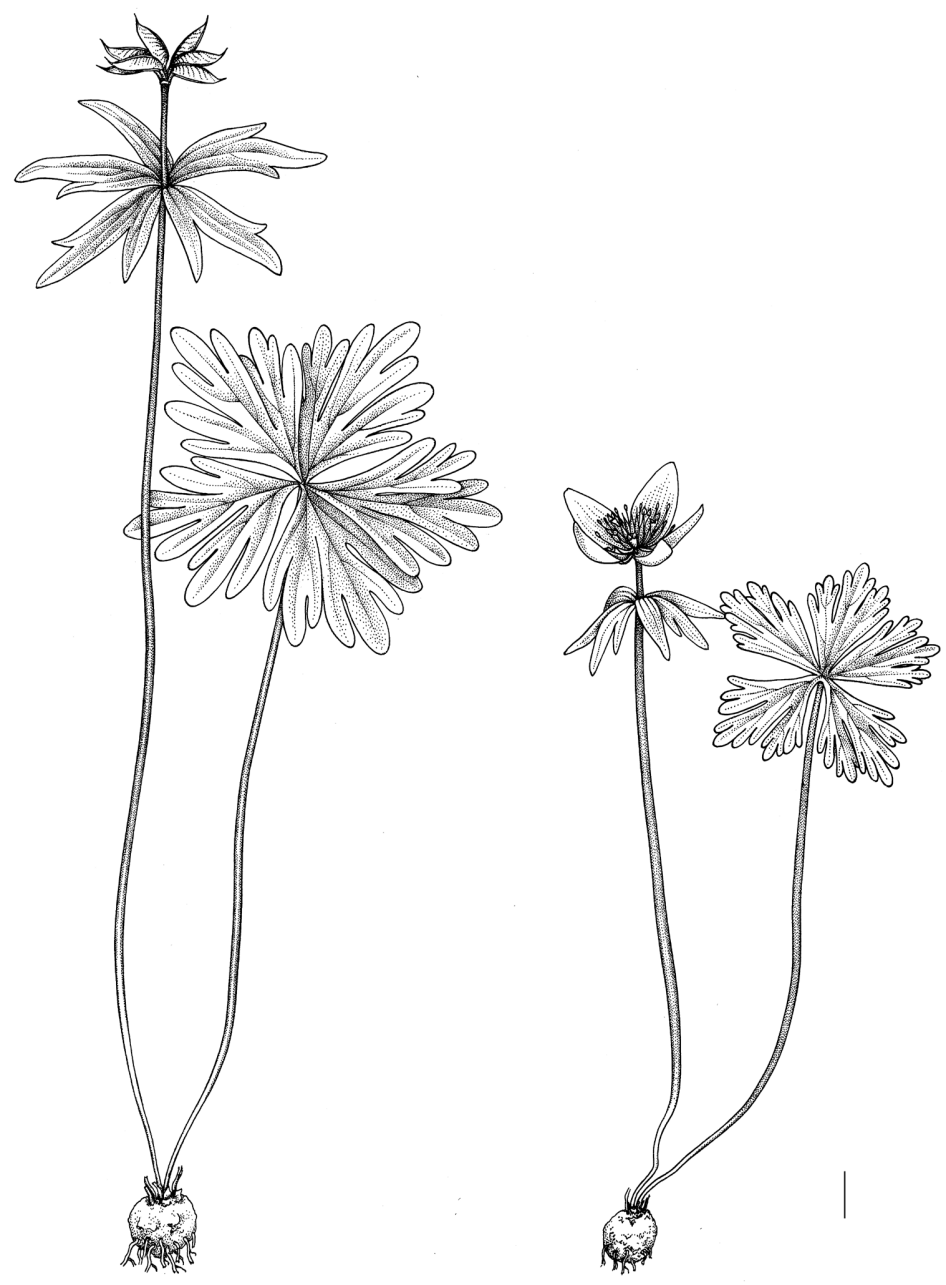
General habit of *Eranthis sibirica*. Scale bar: 1 cm.

**Figure 9.**
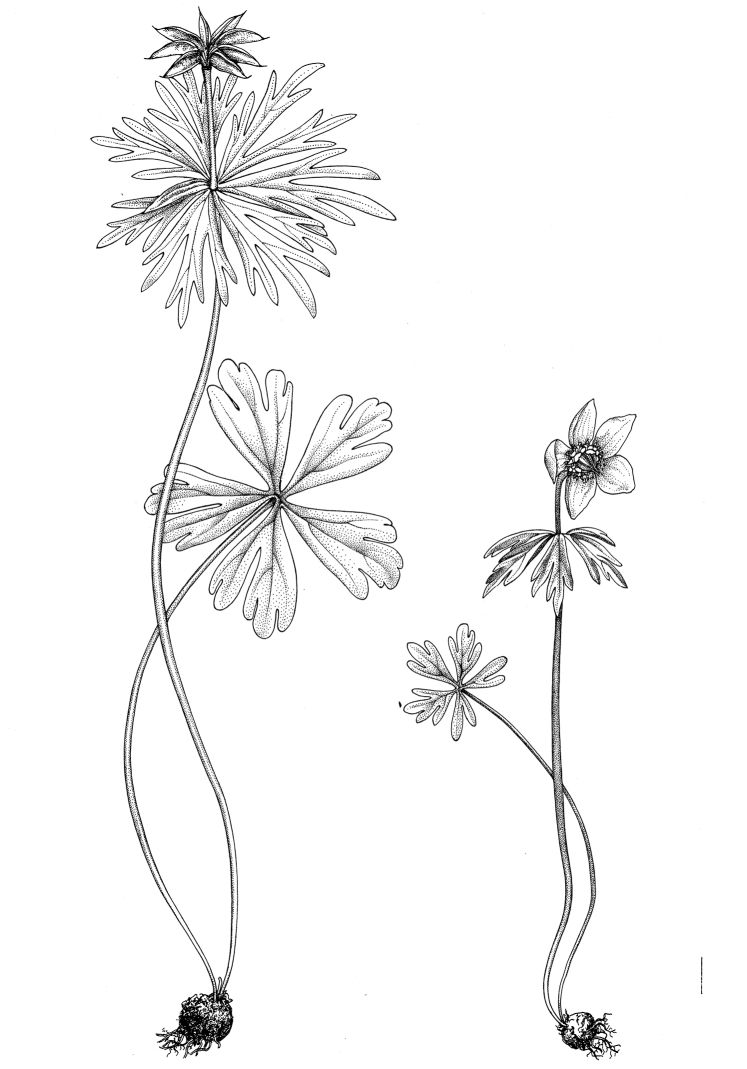
General habit of *Eranthis stellata*. Scale bar: 1 cm.

The new species belongs to E. sect. Shibateranthis (Nakai) Tamura and it is sister to *E. sibirica*, according to the results of molecular phylogenetic analysis (Fig. 1). *E. tanhoensis* is morphologically similar to *E. sibirica* and *E. stellata* (Figs 5–9) in having white sepals, tubular two-lipped petals with bilobate or forked lips, apically acute lobes with abaxial lip and globular yellow swellings (nectaries) at the top or in the central part. The differences amongst the three species are presented in Table 2.

**Table 2.** Morphological differences among *E. sibirica*, *E. tanhoensis*, and *E. stellata*.

Character	*E. sibirica*	*E. tanhoensis*	*E. stellata*
Leaf colour at flowering	green	green	coppery or green
Teeth at the apex of basal leaf segments	rounded	acute	rounded
Maximum dissection of the basal leaf segments (at flowering), cm	1.0	2.3	0.4–?
Maximum dissection of the basal leaf segments (at fruiting), cm	2.3	3.5	1.3
Number of teeth on the segments of the basal leaf (at fruiting)	3–12	6–25	3–5
Apex of involucral leaves	rounded	acute	rounded
Width of the involucral leaf segments (at fruiting), cm	0.4–1.2	1.4–5.3	0.5–2.3
Maximum dissection of the involucral leaf segments (at flowering), cm	1.6	1.6	1.0
Maximum dissection of the involucral leaf segments (at fruiting), cm	2.1	4.0	1.7
Number of teeth on the segments of the involucral leaf (at flowering)	1–5	5–21	3–9
Number of teeth on the segments of the involucral leaf (at fruiting)	2–5	5–21	3–8
Flower position	erect	erect	recurved
Scape pubescence	glabrous or with papillate trichomes	large hemispherical and papillate trichomes	glandular and stellate trichomes
Sepal number	5–7	4–7	5–8
Shape of petals	narrow urn-shaped	broadly urn-shaped	funnelform
Swellings (nectaries) position	at the apex	at the apex	in medium part
Apex colour of adaxial lip	yellow	yellow	white
Apex colour of abaxial lip	yellow	yellow	white
Margin colour between abaxial and adaxial lips	white	yellow	white
Stamen colour	white	white	violet, pink or white
Stylodium length, cm	0.2–0.5	0.1–0.3	0.2–0.4

**Figure 10.**
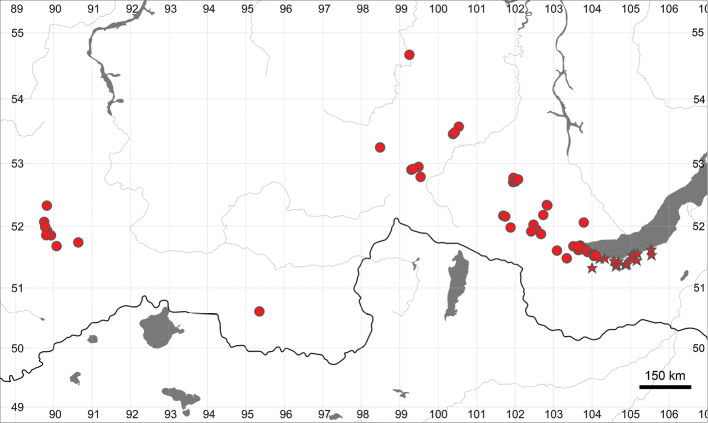
General distribution of *Eranthis sibirica* (dots) and *E. tanhoensis* (stars), based on herbarium materials.

**Figure 11.**
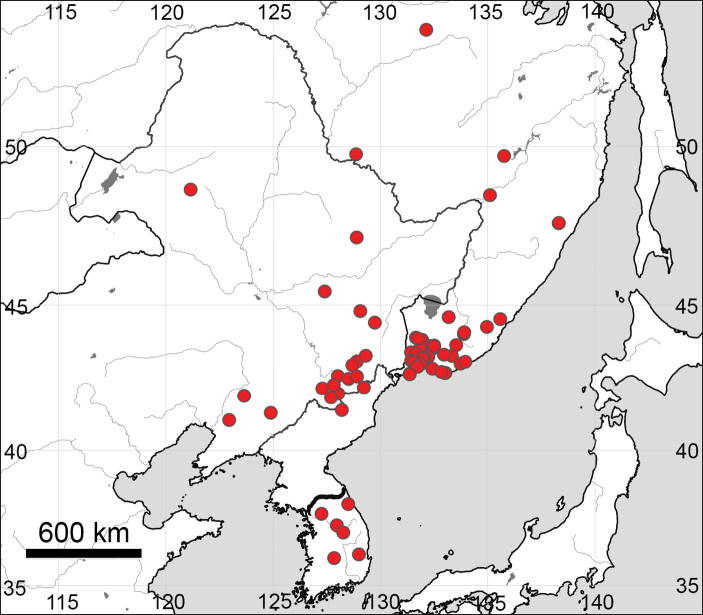
General distribution of *Eranthis stellata*, based on herbarium materials and data in the literature (Oh and Oh 2019; Park et al. 2019).

The new species differs from other related species by dense glandular pubescence of the flower stems, rounded or widely rhombic (not obovate or lanceolate) leaf blade segments, acute, rather than rounded teeth apices of the basal and stem leaves, a large number of teeth and width of the segments of the basal and stem leaves (see also 2). Additionally, all three species growing in Russia have different distribution patterns (Figs 10, 11).

##### Phenology.

Flowering time: April–early May; fruiting time: late May–June.

##### Distribution

(Fig. 10): *Eranthis tanhoensis* is endemic to southern Baikal (Khamar-Daban range of the Republic of Buryatia and Irkutsk Province).

##### Habitat and ecology.

*Eranthis tanhoensis* can be found at 350–2400 m a.s.l., where it grows in fir, Siberian pine, spruce and birch forests, on riverbanks, beside streams (up to 1500 m a.s.l.) and in subalpine meadows (at higher altitudes).

##### Etymology.

The specific epithet of the new species is derived from the type locality, Tanhoi village, Republic of Buryatia, Russia.

##### Additional specimens examined.

Russia: Republic of Buryatia: Kabansky district, Osinovka river (Tanhoi village), 51°33'06.2"N, 105°05'34.7"E, 458 m a.s.l., 20 Jun 2019, *A.S. Erst*, *D.A. Krivenko*, *E.Yu. Mitrenina & O.A. Chernysheva s.n.* (NS-0000949!); Kabansky district, Mishikha river, 51°37'46.7"N, 105°32'05.2"E, 480 m a.s.l., 01 May 2019, *A.S. Erst*, *D.A. Krivenko* & *O.A. Chernysheva 31* (NS-0000950!); Kabansky district, Mishikha river, 51°37'46.7"N, 105°32'05.2"E, 480 m a.s.l., 01 May 2019, *A.S. Erst*, *D.A. Krivenko & O.A. Chernysheva 31a* (NS-0000951!); Kabansky district, Mishikha river, 51°37'32.6"N, 105°32'03.4"E, 478 m a.s.l., 20 Jun 2019, *A.S. Erst*, *D.A. Krivenko*, *E.Yu. Mitrenina & O.A. Chernysheva s.n.* (NS-0000952!); Kabansky district, Dulikha river, 51°32'04.9"N, 105°01'43.2"E, 461 m a.s.l., 01 May 2019, *A.S. Erst*, *D.A. Krivenko & O.A. Chernysheva 14* (NS-0000953!); Kabansky district, Dulikha river, 51°32'04.9"N, 105°01'43.2"E, 461 m a.s.l., 20 Jun 2019, *A.S. Erst*, *D.A. Krivenko*, *E.Yu. Mitrenina & O.A. Chernysheva* (NS-0000954!); Kabansky district, Shestipalikha river, 51°32'46.4"N, 105°04'28.9"E, 465 m a.s.l., 01 May 2019, *A.S. Erst*, *D.A. Krivenko & O.A. Chernysheva s.n.* (NS-0000955!); Kabansky district, Shestipalikha river, 51°32'46.4"N, 105°04'28.9"E, 465 m a.s.l, 21 Jun 2019, *A.S. Erst*, *D.A. Krivenko*, *E.Yu. Mitrenina & O.A. Chernysheva* (NS-0000956!); Kabansky district, Tolbazikha river, 51°26'21.06"N, 104°41'09.82"E, 471 m a.s.l., 02 May 2019, *A.S. Erst*, *D.A. Krivenko & O.A. Chernysheva s.n.* (NS-0000957!); Kabansky district, Tolbazikha river, 51°26'21.06"N, 104°41'09.82"E, 471 m a.s.l., 20 Jun 2019, *A.S. Erst*, *D.A. Krivenko*, *E.Yu. Mitrenina & O.A. Chernysheva s.n.* (NS-0000958!); Irkutsk Province: Slyudyansky district, Semirechka river, 51°28'56.92"N, 104°19'43.47"E, 470 m a.s.l., 02 May 2019, *A.S. Erst*, *D.A. Krivenko & O.A. Chernysheva 048* (NS-0000959!); Slyudyansky district, Semirechka river, 51°28'56.92"N, 104°19'43.47"E, 470 m a.s.l., 21 Jun 2019, *A.S. Erst*, *D.A. Krivenko*, *E.Yu. Mitrenina & O.A. Chernysheva s.n.* (NS-0000960!).

##### Preliminary conservation status.

Although the species seems to have a small distribution area in southern Baikal Lake, the populations observed in 2018 and 2019 consisted of numerous individuals producing viable fruits and no threats to the habitats were observed in the field studies. The EOO of *E. tanhoensis* was estimated for an area of more than 1372 km^2^, while the AOO was 72 km^2^. Preliminary conservation status, according to IUCN’s Extent of Occurrence criteria indicates the species as Endangered (EN) (IUCN 2019).

### Key to the *Eranthis* species growing in Asiatic Russia

**Table:** 

1	Maximum dissection of basal leaf segments ~0.4 cm long at flowering stage, 1.3 cm long at fruiting stage; scape with stellate hairs; involucral leaves green or coppery at flowering; maximum dissection of the involucral leaves 1.7 cm long at fruiting; flowers recurved; petals narrowly funnelform, swellings (nectaries) located in medium part of adaxial lip lobes, apex of abaxial and adaxial lips white; anthers violet, pink or white	***E. stellata***
–	Maximum dissection of basal leaf segments at least 1.0 cm long at flowering, 2.3 cm long at fruiting stage; scape without stellate hairs; involucral leaves green at flowering; maximum dissection of the involucral leaves 2.1 cm long or more at fruiting; flowers erect, petals urn-shaped, swellings (nectaries) located at the apex of adaxial lip lobes, apex of abaxial and adaxial lips yellow; anthers white	**2**
2	Apex of basal and involucral leaves rounded; maximum dissection of basal leaf segments 1.0 cm long at flowering and 2.3 cm long at fruiting; segments of involucral leaves at fruiting 0.4–1.2 cm wide; maximum dissection of the involucral leaves at fruiting 2.1 cm long; each segment of involucral leaves with 1–5 teeth; scape glabrous or papillate; petals narrowly urn-shaped, margins between abaxial and adaxial lips white	***E. sibirica***
–	Apex of basal and involucral leaves acute; maximum dissection of basal leaf segments 2.3 cm long at flowering and 3.5 cm long at fruiting; segments of involucral leaves at fruiting 1.4–5.3 cm wide; maximum dissection of the involucral leaves at fruiting 4.0 cm long; each segment of involucral leaves with 5–21 teeth; scape papillate and with large hemispherical glands; petals broadly urn-shaped, margins between abaxial and adaxial lips yellow	***E. tanhoensis***

## Supplementary Material

XML Treatment for Eranthis tanhoensis

## Supplementary materials

Supplementary material 1 Tables S1–S4 Data type: measurement. Explanation note: **Table S1.** List of samples characters used in molecular (M), cytogenetical (C) and biochemical (B) analyses. **Table S2.** Morphological characters of Russian *Eranthis* species. An asterisk indicates characters used in the numerical analysis. **Table S3.** The results of the variance analysis for plant characters in the flowering stage. The values in parentheses are adjusted P-values; the characters in bold are those without significant interspecific differences. **Table S4.** The results of the variance analysis for plant characters in the fruiting stage. The values in parentheses are adjusted P-values; the characters in bold are those without significant interspecific differences. https://binary.pensoft.net/file/385102This dataset is made available under the Open Database License (http://opendatacommons.org/licenses/odbl/1.0/). The Open Database License (ODbL) is a license agreement intended to allow users to freely share, modify, and use this Dataset while maintaining this same freedom for others, provided that the original source and author(s) are credited.Andrey S. Erst, Alexander P. Sukhorukov, Elizaveta Yu. Mitrenina, Mikhail V. Skaptsov, Vera A. Kostikova, Olga A. Chernisheva, Victoria Troshkina, Maria Kushunina, Denis A. Krivenko, Hiroshi Ikeda, Kunli Xiang, Wei Wang
