# Supplementary material for: An integrative taxonomic approach reveals a new species of Eranthis (Ranunculaceae) in North Asia
Source: PhytoKeys. 2020 Mar 4;140:75–100. doi: 10.3897/phytokeys.140.49048 (PMC7066265; doi:10.3897/phytokeys.140.49048)
Supplement: Supplementary material 1 [file phytokeys-140-075-s001.docx]

**Table S1. List of samples characters used in molecular (M), cytogenetical (C) and biochemical (B) analyses.**

| **№** | **Taxon** | **Collector number** | **Locality** | **Coordinates, elevation** | **Сollector(s), date** | **Type of analysis** |
| --- | --- | --- | --- | --- | --- | --- |
| 1 | *E. sibirica* | a4-122 | Russia, Republic of Khakassia, Tashtypsky District, Bolshoi On village, Bolshoi On river | 52°04'02.1"N 89°45'33.6"E | *A. Erst, T. Erst*, 04.05.2018 | С |
| 2 | *E. sibirica* | a5-539 | Russia, Irkutsk province, Slyudyansky District, Burovshina river | 51°37'06.00''N, 103°49'16.17''E  alt. 470 m | *A.S. Erst, D.A. Krivenko, O.A. Chernysheva*, 02.05.2019 | M, C, B |
| 3 | *E. sibirica* | a5-568 | Russia, Irkutsk province, Slyudyansky District, Burovschina river | 51°37'12.2"N 103°49'22.3"E | *A.A. Kiseleva*, 25.06.1982 | M |
| 4 | *E. sibirica* | a5-541 | Russia, Irkutsk province, Slyudyansky District, Utulik river | 51°32'50''N, 104°02'45''E  alt. 464 m | *A.S. Erst, D.A. Krivenko, O.A.* Chernysheva, 02.05.2019 | M, C, B |
| 5 | *E. sibirica* | a5-555 | Russia, Irkutsk Province, Slyudyansky District, Utulik river | 51°31'15''N, 104°02'58''E  alt. 525 m | *S.G. Kazanovsky*, 14.06.2011 | M |
| 6 | *E. sibirica* | a5-574 | Russia, Irkutsk Province, Slyudyansky District, valley of Slyudyanka river | 51°37'01''N, 103°38'37''E | *A.V. Verhozina, V.V. Chepinoga*, 21.06.2001 | M |
| 7 | *E. sibirica* | a5-542 | Russia, Irkutsk Province, Slyudyansky District, vicinity of Slyudyanka town | 51°38'02.94''N, 103°41'13.90''E  alt. 531 m | *A.S. Erst, D.A. Krivenko, O.A. Chernysheva*, 02.05.2019 | C, B |
| 8 | *E. sibirica* | a5-553 | Russia, Irkutsk Province, Slyudyansky District, vicinity of Slyudyanka town | 51°37'41''N, 103°41'27''E | *N.V.* Stepantsova, 27.04.1996 | M |
| 9 | *E. sibirica* | a4-123 | Russia, Irkutsk Province, Shelikhovsky District, Kuitun river, Shamanka village | 52°03'39.2"N 103°47'01.3"E  alt. 492 m | *A.S. Erst, E. Yu. Mitrenina, D.A. Krivenko, O.A. Chernysheva*, 20.05.2019 | С |
| 10 | *E. tanhoensis* | a6-121 | Russia, Irkutsk Province, Kabansky District, Mamai river | 51°26'52.0''N, 104°49'01.3''E  alt. 485 m | *A.S. Erst, E.Yu. Mitrenina, D.A. Krivenko, O.A. Chernysheva*, 20.06.2019 | C |
| 11 | *E. tanhoensis* | a5-532 | Russia, Republic of Buryatia, Kabansky District, Mishikha river | 51°37'46.7''N, 105°32'05.2''E  alt. 480 m | *A.S. Erst, D.A. Krivenko, O.A. Chernysheva*, 01.05.2019 | M, C, B |
| 12 | *E. tanhoensis* | a5-533 | Russia, Republic of Buryatia, Kabansky District, Mishikha river | 51°37'32.6''N, 105°32'03.4''E  alt. 478 m | *A.S. Erst, D.A. Krivenko, O.A. Chernysheva*, 01.05.2019 | M, C, B |
| 13 | *E. tanhoensis* | a5-570 | Russia, Republic of Buryatia, Kabansky District, Mishikha river | 51°19'03.0"N 105°26'32.0"E | *M.M. Ivanova*, 21.07.1963 | M |
| 14 | *E. tanhoensis* | a5-534 | Russia, Republic of Buryatia, Kabansky District, Dulikha river | 51°32'04.9''N, 105°01'43.2''E  alt. 461 m | *A.S. Erst, D.A. Krivenko, O.A. Chernysheva*, 01.05.2019 | M, C, B |
| 15 | *E. tanhoensis* | a5-535 | Russia, Republic of Buryatia, Kabansky District, Shestipalikha river | 51°32'46.4''N, 105°04'28.9''E  alt. 465 m | *A.S. Erst, D.A. Krivenko, O.A. Chernysheva*, 01.05.2019 | M, C, B |
| 16 | *E. tanhoensis* | a5-536 | Russia, Republic of Buryatia, Kabansky District, Osinovka river (Tanhoi village) | 51°33'06.2''N, 105°05'34.7''E  alt. 458 m | *A.S. Erst, D.A. Krivenko, O.A. Chernysheva*, 01.05.2019 | M, C, B |
| 17 | *E. tanhoensis* | a5-572 | Russia, Republic of Buryatia, Kabansky District, Tanhoi village, Pereemnaya river | 51°33'40''N, 105°10'33''E | *S.G. Kazanovsky*, 02.05.2003 | M |
| 18 | *E. tanhoensis* | a5-573 | Russia, Republic of Buryatia, Kabansky District, Tanhoi village, Pereemnaya river | 51°33'37''N, 105°10'29''E | *S.G. Kazanovsky*, 02.05.2003 | M |
| 19 | *E. tanhoensis* | a3-388 | Russia, Republic of Buryatia, Kabansky District, Osinovka river | 51°45'N,  105°16'E  alt. 481 m | *N. Gamova*, 15.06.2012 | M |
| 20 | *E. tanhoensis* | a5-537 | Russia, Republic of Buryatia, Kabansky District, Tolbazikha river | 51°26'21.06''N, 104°41'09.82''E  alt. 471 m | *A.S. Erst, D.A. Krivenko, O.A. Chernysheva*, 01.05.2019 | M, C, B |
| 21 | *E. tanhoensis* | a5-538 | Russia, Irkutsk Province, Slyudyansky District, Semirechka river | 51°28'56.92''N, 104°19'43.47''E  alt. 470 m | *A.S. Erst, D.A. Krivenko, O.A. Chernysheva*, 01.05.2019 | M, C, B |
| 22 | *E. tanhoensis* | a5-554 | Russia, Irkutsk Province, Slyudyansky District, Taltsinka river, left bank of Snezhnaya river | 51°21'26''N, 104°37'04''E | *N.V. Stepantsova*, 23.06.2003 | M |
| 23 | *E. tanhoensis* | a5-556 | Russia, Irkutsk Province, Slyudyansky District, Solzan river | 51°28'51''N, 104°11'53''E | *M.M. Ivanova*, 17.05.2006 | M |
| 24 | *E. tanhoensis* | a5-564 | Russia, Irkutsk Province, Slyudyansky District, Taltsy river | 51°22'35.2"N 104°38'44.3"E | *S.G. Kazanovsky*, 31.07.1994 | M |
| 25 | *E. tanhoensis* | a5-571 | Russia, Republic of Buryatia, Kabansky District, Anosovka and Right Anosovka river | 51°28'27.0"N 105°00'46.0"E | *S.G. Kazanovsky*, 22.06.1995 | M |
| 26 | *E. tanhoensis* | a5-540 | Russia, Irkutsk Province, Slyudyansky District, Malye Mangaly river | 51°26'48.17''N, 104°34'16.62''E  alt. 471 m | *A.S. Erst, D.A. Krivenko, O.A. Chernysheva*, 02.05.2019 | M, C, B |
| 27 | *E. stellata* | a5-558 | Russia, Primorskiy Krai, Vladivostok city, Sanatornaya railway station | 43°13'39.5"N, 131°58'44.7"E | *S. Kharkevich, T. Buch*, 23.04.1995 | M |
| 28 | *E. stellata* | a5-551 | Russia, Primorsky Krai, Nakhodka town, Kozmino village | 42°43'16.9"N 133°01'32.9"E | *S.G. Kazanovsky*, 06.06.2009 | M |
| 29 | *E. stellata* | a6-100 | Russia, Primorsky Krai, Vladivostok city, Akademicheskaya land transport station | 43°11'20.4"N,  131°55'25.4"E  alt. 471 m | *V. Yakubov*, 12.04.2018 | С |
| 30 | *E. stellata* | a6-101 | Russia, Primorsky Krai, Vladivostok city, 13th km railway station | 43°11'32.3"N, 131°55'49.0"E | *V. Nikulin, A. Nikulinn*, 14.05.2018 | C, B |
| 31 | *E. stellata* | a6-102 | Russia, Primorsky Krai, Vladivostok city, Malaya Sedanka river | 43°12'37.9"N 131°59'39.1"E | *V. Nikulin, A. Nikulin*, 16.05.2018 | C, B |
| 32 | *E. stellata* | a6-103 | Russia, Primorsky Krai, Vladivostok city, Russky Island | 42°59'05.0"N 131°51'51.5"E | *V. Nikulin, A.* Nikulin, 12.05.2018 | C, B |
| 33 | *E. pinnatifida* | a5-547 | Japan, Mie Prefecture, Hokuseicho Betsumyo, Inabe City | 35°08'22.6"N 136°28'20.0"E | *A.S. Erst, T.V. Erst, Ikeda H. et al.*  03.04.2019 | M |
| 34 | *E. longistipitata* | a5-529 | Kyrgistan, Chuya Province, near Bishkek City, Bozbelbek Village | 42°46'1"N 74°34'48"E | *E.V. Boltenkov, S.N. Zenin*  20.03.2019 | M |

**Table S2.** **Morphological characters of Russian *Eranthis* species*.*** Asterisk indicates characters used in numeric analysis.

| **Number of character** | **Character** | ***E. sibirica*** | ***E*. *tanhoensis*** | ***E. stellata*** |
| --- | --- | --- | --- | --- |
| 1* | Plant height (at flowering), cm | 13.7–26.3 | 12.8–22.7 | 8.9–21.8 |
| 2* | Plant height (at fruiting), cm | 21.4–38.1 | 18.2–39.2 | 14.4–33.0 |
| 3 | Leaves colour at flowering | green | green | green or coppery |
| 4 | Teeth at the apex of basal leaf segments | rounded | acute | rounded |
| 5* | Length of the basal leaf segments (at flowering), cm | 1–2.5 | 0.8–2.5 | 1.2–? |
| 6* | Length of the basal leaf segments (at fruiting), cm | 2.4–5.1 | 1.7–8.5 | 1.1–2.7 |
| 7* | Width of the basal leaf segments (at flowering), cm | 0.5–1.7 | 0.4–1.8 | 0.8–? |
| 8* | Width of the basal leaf segments (at fruiting), cm | 1.7–3.3 | 1.2–7.5 | 1.5–2.8 |
| 9* | Maximum dissection of the basal leaf segments (at flowering), cm | 1 | 2.3 | 0.4–? |
| 10* | Maximum dissection of the basal leaf segments (at fruiting), cm | 2.3 | 3.5 | 1.3 |
| 11* | Number of segment lobes on basal leaf (at flowering) | 1–2 | 1–2 | 1–2 |
| 12* | Number of segment lobes on basal leaf (at fruiting) | 1–2 | 1–2 | 1–2 |
| 13* | Number of teeth on a segments of the basal leaf (at flowering) | 5–12 | 6–19 | 6–? |
| 14* | Number of teeth on the segments of the basal leaf (at fruiting) | 3–12 | 6–25 | 3–5 |
| 15 | Apex of involucral leaves | rounded | acute | rounded |
| 16* | Length of the involucral leaf segments (at flowering), cm | 1.3–3.3 | 1.1–3.0 | 0.7–2.4 |
| 17* | Length of the involucral leaf segments (at fruiting), cm | 1.6–5.1 | 3.3–6.4 | 1.8–3.9 |
| 18* | Width of the involucral leaf segments (at flowering), cm | 0.2–0.9 | 0.5–2.5 | 0.2–1.1 |
| 19* | Width of the involucral leaf segments (at fruiting), cm | 0.4–1.2 | 1.4–5.3 | 0.5–2.3 |
| 20* | Maximum dissection of the involucral leaf segments (at flowering), cm | 1.6 | 1.6 | 1.0 |
| 21* | Maximum dissection of the involucral leaf segments (at fruiting), cm | 2.1 | 4.0 | 1.7 |
| 22* | Number of segment lobes on the involucral leaf (at flowering) | 1–2 | 2 | 1 |
| 23* | Number of segment lobes on the involucral leaf (at fruiting) | 1–2 | 2 | 2 |
| 24* | Number of teeth on segment of the involucral leaf (at flowering) | 1–5 | 5–21 | 3–9 |
| 25* | Number of teeth on segment of the involucral leaf (at fruiting) | 2–5 | 5–21 | 3–8 |
| 26 | Flower position | erect | erect | recurved |
| 27 | Scape pubescence | glabrous or with papillate trichomes | large hemispherical and papillate trichomes | glandular and stellate trichomes |
| 28* | Sepal number | 5–7 | 4–7 | 5–8 |
| 29* | Sepal length, cm | 0.9–2.2 | 1.1–2.6 | 1.1–1.6 |
| 30* | Sepal width, cm | 0.6–1.2 | 0.5–1.3 | 0.2–1.0 |
| 31* | Petal number | 6–10 | 5–15 | 10–14 |
| 32* | Petal length, cm | 0.6–0.7 | 0.6–0.8 | 0.5–0.7 |
| 33 | Shape of petals | narrow urn-shaped | broadly urn-shaped | funnelform |
| 34 | Swellings (nectaries) position | at the apex | at the apex | in medium part |
| 35 | Apex colour of adaxial lip | yellow | yellow | white |
| 36 | Apex colour of abaxial lip | yellow | yellow | white |
| 37 | Margin colour between abaxial and adaxial lips | white | yellow | white |
| 38 | Stamen colour | white | white | violet, pink or white |
| 39* | Follicle number | 3–9 | 3–10 | 3–13 |
| 40* | Follicle length, cm | 0.8–1.8 | 0.8–1.4 | 1.0–1.5 |
| 41 | Stylodium length, cm | 0.2–0.5 | 0.1–0.3 | 0.2–0.4 |

**Table S3.** **The results of the variance analysis for plant characters at flowering stage.** The values in parentheses are adjusted P-values, the characters in bold are those without significant interspecific differences.

| **Character number** | **Character** | **F-value** | **P-value** | **Species** | **Character mean value** |
| --- | --- | --- | --- | --- | --- |
| 1 | Plant height (at flowering), cm | 7.878 | 0.000798  (0.01197) | *E. sibirica* | 18.884 |
|  |  |  |  | *E. tanhoensis* | 16.43077 |
|  |  |  |  | *E. stellata* | 15.464 |
| 5 | **Length of the basal leaf segments (at flowering), cm** | **3.469** | **0.0364**  **(0.546)** | *E. sibirica* | 1.742 |
|  |  |  |  | *E. tanhoensis* | 1.790385 |
|  |  |  |  | *E. stellata* | 1.519797 |
| 7 | **Width of the basal leaf segments (at flowering), cm** | **5.223** | **0.00759**  **(0.11385)** | *E. sibirica* | 1.04 |
|  |  |  |  | *E. tanhoensis* | 1.103846 |
|  |  |  |  | *E. stellata* | 0.8466807 |
| 9 | Maximum dissection of the basal leaf segments (at flowering), cm | 10.58 | >0.000  (0.0013) | *E. sibirica* | 0.678 |
|  |  |  |  | *E. tanhoensis* | 0.4461538 |
|  |  |  |  | *E. stellata* | 0.5439631 |
| 13 | **Number of teeth on the segments of the basal leaf (at flowering)** | **3.911** | **0.0243**  **(0.3645)** | *E. sibirica* | 7.76 |
|  |  |  |  | *E. tanhoensis* | 9.461538 |
|  |  |  |  | *E. stellata* | 8.37147 |
| 16 | Length of the involucral leaf segments (at flowering), cm | 23.38 | >0.000  (>0.000) | *E. sibirica* | 2 |
|  |  |  |  | *E. tanhoensis* | 2.480769 |
|  |  |  |  | *E. stellata* | 1.472 |
| 18 | Width of the involucral leaf segments (at flowering), cm | 30.93 | >0.000  (>0.000) | *E. sibirica* | 0.448 |
|  |  |  |  | *E. tanhoensis* | 1.196154 |
|  |  |  |  | *E. stellata* | 0.624 |
| 20 | **Maximum dissection of the involucral leaf segments (at flowering), cm** | **1.304** | **0.278**  **(1)** | *E. sibirica* | 0.728 |
|  |  |  |  | *E. tanhoensis* | 0.6384615 |
|  |  |  |  | *E. stellata* | 0.596 |
| 22 | Number of segment lobes on the involucral leaf (at flowering) | 13.78 | >0.000  (0.00012) | *E. sibirica* | 1.64 |
|  |  |  |  | *E. tanhoensis* | 2 |
|  |  |  |  | *E. stellata* | 2 |
| 24 | Number of teeth on segment of the involucral leaf (at flowering) | 61.2 | >0.000  (>0.000) | *E. sibirica* | 2.76 |
|  |  |  |  | *E. tanhoensis* | 10.23077 |
|  |  |  |  | *E. stellata* | 4.96 |
| 28 | **Sepal number** | **0.38** | **0.685**  **(1)** | *E. sibirica* | 5.28 |
|  |  |  |  | *E. tanhoensis* | 5.346154 |
|  |  |  |  | *E. stellata* | 5.44 |
| 29 | Sepal length, cm | 51.14 | >0.000  (>0.000) | *E. sibirica* | 1.822 |
|  |  |  |  | *E. tanhoensis* | 1.796154 |
|  |  |  |  | *E. stellata* | 1.276 |
| 30 | Sepal width, cm | 27.74 | >0.000  (>0.000) | *E. sibirica* | 0.876 |
|  |  |  |  | *E. tanhoensis* | 0.8115385 |
|  |  |  |  | *E. stellata* | 0.572 |
| 31 | Petal number | 13.99 | >0.000  (0.000108) | *E. sibirica* | 8.6 |
|  |  |  |  | *E. tanhoensis* | 9.961538 |
|  |  |  |  | *E. stellata* | 11.12 |
| 32 | Petal length, cm | 29.5 | >0.000  (>0.000) | *E. sibirica* | 0.648 |
|  |  |  |  | *E. tanhoensis* | 0.6326923 |
|  |  |  |  | *E. stellata* | 0.54 |

**Table S4.** **The results of the variance analysis for plant characters at fruiting stage.** The values in parentheses are adjusted P-values, the characters in bold are those without significant interspecific differences.

| **Character number** | **Character** | **F-value** | **P-value** | **Species** | **Character mean value** |
| --- | --- | --- | --- | --- | --- |
| 2 | **Plant height (at fruiting), cm** | **4.045** | **0.0216**  **(0.324)** | *E. sibirica* | 29.596 |
|  |  |  |  | *E. tanhoensis* | 26.63077 |
|  |  |  |  | *E. stellata* | 25.942 |
| 6 | **Length of the basal leaf segments (at fruiting), cm** | **2.187** | **0.12**  **(1)** | *E. sibirica* | 4 |
|  |  |  |  | *E. tanhoensis* | 4.111538 |
|  |  |  |  | *E. stellata* | 3.586904 |
| 8 | Width of the basal leaf segments (at fruiting), cm | 0.69 | 0.505  (1) | *E. sibirica* | 2.37 |
|  |  |  |  | *E. tanhoensis* | 2.55 |
|  |  |  |  | *E. stellata* | 2.457519 |
| 10 | Maximum dissection of the basal leaf segments (at fruiting), cm | 15.38 | >0.000  (>0.000) | *E. sibirica* | 1.64 |
|  |  |  |  | *E. tanhoensis* | 1.153846 |
|  |  |  |  | *E. stellata* | 1.334328 |
| 14 | Number of teeth on the segments of the basal leaf (at fruiting) | 17.15 | >0.000  (>0.000) | *E. sibirica* | 8.16 |
|  |  |  |  | *E. tanhoensis* | 13.07692 |
|  |  |  |  | *E. stellata* | 10.04862 |
| 17 | Length of the involucral leaf segments (at fruiting), cm | 23.0 | >0.000  (>0.000) | *E. sibirica* | 3.448 |
|  |  |  |  | *E. tanhoensis* | 4.296154 |
|  |  |  |  | *E. stellata* | 2.8 |
| 19 | Width of the involucral leaf segments (at fruiting), cm | 61.53 | >0.000  (>0.000) | *E. sibirica* | 0.764 |
|  |  |  |  | *E. tanhoensis* | 2.221154 |
|  |  |  |  | *E. stellata* | 1.42 |
| 21 | **Maximum dissection of the involucral leaf segments (at fruiting), cm** | **2.557** | **0.0845**  **(1)** | *E. sibirica* | 1.34 |
|  |  |  |  | *E. tanhoensis* | 1.288462 |
|  |  |  |  | *E. stellata* | 1.104 |
| 23 | Number of segment lobes on the involucral leaf (at fruiting) | 9.525 | 0.000211  (0.0003165) | *E. sibirica* | 1.72 |
|  |  |  |  | *E. tanhoensis* | 2 |
|  |  |  |  | *E. stellata* | 2 |
| 25 | Number of teeth on segment of the involucral leaf (at fruiting) | 77.35 | >0.000  (>0.000) | *E. sibirica* | 3.12 |
|  |  |  |  | *E. tanhoensis* | 11.38462 |
|  |  |  |  | *E. stellata* | 5.2 |
| 39 | **Follicle number** | **4.55** | **0.0137**  **(0.2055)** | *E. sibirica* | 5.96 |
|  |  |  |  | *E. tanhoensis* | 5.192308 |
|  |  |  |  | *E. stellata* | 6.68 |
| 40 | Follicle length, cm | 6.533 | 0.00245  (0.03675) | *E. sibirica* | 1.324 |
|  |  |  |  | *E. tanhoensis* | 1.119231 |
|  |  |  |  | *E. stellata* | 1.268 |
| 41 | Stylodium length, cm | 44.76 | >0.000  (>0.000) | *E. sibirica* | 0.346 |
|  |  |  |  | *E. tanhoensis* | 0.1923077 |
|  |  |  |  | *E. stellata* | 0.264 |
